# Healthy Madrasas: a qualitative study using normalisation process theory and co-production approach to explore translation of a childhood obesity prevention intervention in Islamic religious settings from one UK city to another

**DOI:** 10.1136/bmjopen-2025-115161

**Published:** 2026-06-29

**Authors:** Elin Cawley, Russell Jago, Samina Baig, Sufyan A Dogra, Zia Haque, Julian Hamilton-Shield, Sally E Barber, Nour Al Husein

**Affiliations:** 1 NIHR Bristol Biomedical Research Centre, University Hospitals Bristol and Weston NHS Foundation Trust and University of Bristol, Bristol, UK; 2 Population Health Sciences, Bristol Medical School, University of Bristol, Bristol, UK; 3 Caafi Health, Bristol, UK; 4 Bradford Institute for Health Research, Bradford, UK; 5 Translational Health Sciences, Bristol Medical School, University of Bristol, Bristol, UK

**Keywords:** Obesity, Preventive Health Services, Community-Based Participatory Research, Community child health, Islam, Exercise

## Abstract

**Abstract:**

**Background:**

Childhood obesity remains high in the UK, with a higher prevalence among children from ethnic minority groups. The ‘Healthy Madrasa’ programme was developed and implemented in Bradford, UK, to support obesity prevention in Muslim children by working with Islamic religious settings such as madrasas.

**Objectives:**

The aim of this study was to explore how the ‘Healthy Madrasa’ programme could be adapted for delivery in another UK city where the proportion of people from the Muslim faith is lower and the ethnic composition is more diverse.

**Design:**

A qualitative study involving a co-production workshop and eight focus groups was conducted utilising topic guides guided by the normalisation process theory (NPT). Data were analysed thematically using NVivo software (V.15) and mapped onto the constructs of the NPT.

**Setting:**

Bristol City, UK (2024–2025).

**Participants:**

34 participants took part, including representatives of Islamic organisations, voluntary sector organisations, public health experts and Muslim parents. Data were collected through one co-production workshop (n=15, 47% female) and eight focus groups (n=19, 100% female) of which two were conducted as individual interviews due to single attendance (n=1).

**Results:**

Three constructs of the NPT have been identified. Participants showed positive engagement towards the programme ‘cognitive participation, NPT’, they highlighted an equity gap in current provision due to issues of inclusivity, safe space and the lack of culturally appropriate opportunities for Muslim girls ‘coherence, NPT’. However, barriers to ‘coherence, NPT’ were noted by Muslim parents, stating that cultural and religious conservatism might hinder female participation in the programme. ‘Collective action, NPT’, included the practicalities of transferring the programme to Bristol and assigning the roles and responsibilities. Participants emphasised the importance of creating an ‘Islamic community-led’ steering group, inclusive of diverse communities, building effective partnerships with other relevant organisations and supporting the programme with sufficient resources, such as culturally acceptable funding, space and volunteers.

**Conclusions:**

This study generated a co-produced preliminary roadmap for implementation. Further collaboration with stakeholders is required to achieve coherence and collective action before the programme’s operationalisation in Bristol.

**STRENGTHS AND LIMITATIONS OF THIS STUDY**

- The study was supported by local health ambassadors. Their involvement was vital in building trust and relationships with the communities, thereby enhancing the relevance and credibility of our research findings.
- No male participants took part in the parent focus groups. Thus, the perspectives of fathers on the programme were not captured.
- Most of our participants were of South Asian heritage. We had limited participation from the other Islamic communities within Bristol.

## Introduction

 The prevalence of childhood obesity in the UK is high, with 13% of school year 6 children (~11 years of age) overweight and 22.2% living with obesity.^1^ A child living in the most deprived areas is more than twice as likely to be obese (29.3%) than a child living in the least deprived areas (13.5%).^1 2^ The prevalence also differs by ethnicity^3^ with obesity affecting 25% of South Asian and 30.6% of black children in year 6 compared with 20.8% of white British children.^1^ These findings suggest that there is a need to find ways to prevent obesity among children from low-income and ethnic minority households in the UK. This will require changes to diet, physical activity (PA) and the surrounding environment, such as neighbourhoods and communities where they spend time after school.

A series of studies have suggested that the ethnic inequality in obesity results from a combination of behavioural factors, including diet and other health behaviours, genetic predisposition and lack of culturally related intentions.^4^,^7^ There is some evidence that South Asian children are less physically active and spend more time sedentary than white British children.^3^ Obesogenic behaviours, such as low fruit and vegetable intake and PA, and greater levels of sedentary time have all been associated with serious chronic illnesses such as cardiovascular disease, coronary heart disease, type 2 diabetes and stroke.^4 8^ Additionally, evidence shows that white populations are more likely to take part in health promoting interventions than ethnic minorities, contesting the assumption that an effective health promotion intervention targeted at the general population can also be effective for ethnic minorities.^9^

Evidence shows that children’s lifestyle habits, such as diet and PA, can be influenced by ‘role models’, such as parents.^10^,^12^ In this regard, recent studies have provided evidence that religious influential figures could play a significant role in supporting and promoting a healthy lifestyle for Muslims.^13 14^ Mosques may provide a space for Muslims to provide health-promoting interventions.^15^ Research conducted in Bradford has shown that religious settings (eg, madrasas) could be a good location within which to deliver obesity prevention programmes for South Asian children.^16^ A madrasa is a traditional religious school where Islamic studies, such as Quran learning, are taught at places of worship like mosques.^17^ Researchers in Bradford developed and implemented a programme called The Healthy Madrasas Programme, which aimed to facilitate organisational and individual behaviour change and address local structural and systematic drivers of physical inactivity and obesity to improve the health and well-being of Muslim children by working with Islamic religious settings, such as the madrasa. The programme specifically addresses making changes to the diet and PA levels of Muslim children and families to support obesity prevention. Thus far, the intervention has been delivered in 27 madrasas, reaching >4500 children across the Bradford district.^18^ Qualitative data in response to this implementation in Bradford showed that Islamic leaders, Islamic religious settings workers and Muslim parents felt supportive towards the programme.

Bradford’s South Asian and Muslim population is unique, with a distinctively higher population of ethnic minority communities (32.1% identified as Asian or British Asian, 2% identified as Black, 30.5% are Muslims) in comparison to the rest of the UK.^19^ Therefore, there is a need to examine how the research from Bradford could be translated to other relevant settings across the UK and what changes might be needed to the programme content to maximise acceptability and feasibility in a non-Bradford setting. This is important as it will increase the programme’s representativeness and allow its implementation in more cities throughout the UK, which may have lower populations of Muslim minority communities and differing countries of Islamic heritage. As such this research aimed to explore how the Healthy Madrasas Programme (developed in Bradford) could be modified to be delivered within another UK city (Bristol) where the proportion of people from the Muslim faith is lower (6.7%) and similar to the national average (6.5%), and the ethnic demographics is different, with many Muslims in Bristol being of Black African, Somali as well as South Asian heritage.^20^,^22^ Specifically, we sought to identify barriers and co-produce recommendations for the integration of an intervention, ‘The Healthy Madrasas Programme’, into madrasas settings in Bristol.

## Methods

### Healthy Madrasas Programme

**Figure 1:**
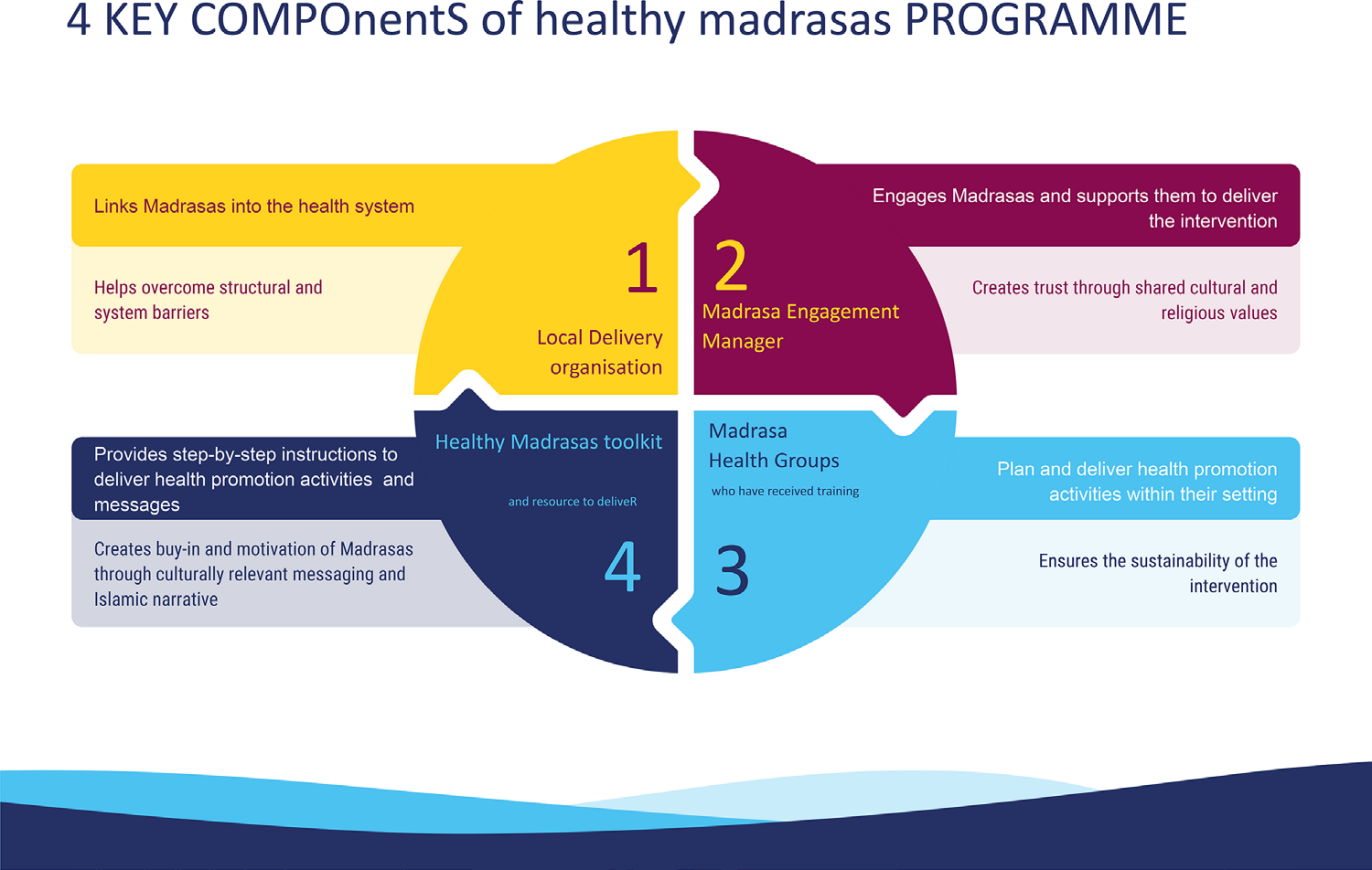
The four key components of the Bradford Healthy Madrasas programmes (copied from reference^25^ with permission).

The Healthy Madrasas Programme in Bradford (figure 1) consists of four key components to the intervention.^23^,^25^ These include: (1) a delivery organisation that coordinates with the madrasas and local leaders. It secures and manages funds to support the intervention’s implementation and employs a team of community engagement managers. (2) Community engagement managers who are trained in organising and delivering sports and PA sessions based on the Healthy Madrasa toolkit.^26^ They have a strong understanding of the madrasas’ cultural and religious values, which helps foster trust. (3) Within each madrasa, a team of volunteers and mosque/madrasa leaders and workers, known as the health group. This group works closely with the community engagement managers to implement the toolkit. (4) The co-produced Healthy Madrasa Toolkit, which provides detailed guidelines on delivering various healthy living, diet, sport and physical activities that are culturally and religiously appropriate.

### Study design and theoretical framework

A qualitative participatory design using multiple methods was employed to co-produce a plan to adapt the Healthy Madrasas Programme, to be suitable for the Bristol context. It comprised a co-production workshop with representatives from organisations within the Muslim community and the voluntary sector, along with focus groups (FGs) and interviews involving parents of children from the Muslim community. Participants were additionally asked to complete a brief questionnaire capturing key demographic information. Co-production methods are believed to produce interventions that reflect participants’ needs and experiences while promoting end users’ ownership of the co-produced services, which may increase intervention effectiveness and user engagement.^27^,^29^ Our study drew on O’Cathain’s ‘Partnership Approach’ to intervention development,^30^ which proposes six steps to guide the co-production approach. The study was also underpinned by the normalisation process theory (NPT): a theoretical framework that helps identify barriers to the uptake of an intervention in a given context, that is, integration of a complex intervention into routine practice.^31 32^ NPT has four constructs: coherence; the work people do to make sense of a new intervention, cognitive participation; rational work around participation (degree of engagement and commitment over time), collective action; the operational work that people do to adopt the intervention including interactions, skills and resources, reflexive monitoring; the appraisal work that people do to feedback and optimise the intervention over time. Table 1 illustrates how the study design aligns with O’Cathain’s six steps of co-production and maps onto the NPT constructs.

**Table 1:** The use of O’Cathain’s six steps in the current study^30 60^

O’Cathain’s six steps	Application in this study and links to NPT
Identify a team of end-users and relevant stakeholders	Bristol Council Public Health representatives Voluntary sector representatives Prominent individuals/organisations within the Muslim community in Bristol Islamic religious school (IRS) teachers Parents
Define and share knowledge and experiences, and understand the current problem	The workshop and focus groups included a presentation on the Bradford model components and the Bradford IRS’ experiences of the model Following that, the discussion was open to explore the barriers to integration into the Bristol context. This was led by the coherence element of the NPT which explores the work people do to make sense of the intervention and its purpose
Co-create the vision by listening to all voices	Participants were invited to envision the programme for the Bristol IRS context. This component was guided by the cognitive participation of the NPT, which aims to promote participation, ownership and maximise engagement
Co-design the solutions using qualitative research	This component was guided by the collective action of the NPT, focusing on the operational work that people do to adopt a set of practices
Build the solution, possibly using small action groups who can use their relevant expertise. Make use of prototyping methods Measure outcomes together and plan this as an integral part of the process	These components build into the collective action and the reflexive monitoring of the NPT. These will be used in future work

NPT, normalisation process theory.

### Patient and public involvement and engagement

During the study, we sought the support of a local community organisation with deep links with members of the Muslim community to support our engagement with the community. This local community organisation hosts a pilot project called the health ambassadors. The health ambassadors programme is a pilot initiative that aims to strengthen connections between researchers, organisations and underserved communities, thereby increasing the body of research addressing health inequalities.^33^ Health ambassadors are members of the public undertaking scientific work in collaboration with professional scientists. Their work often involves outreach and public engagement activities.^34 35^ They also act as community activists and volunteers committed to improving the well-being of the communities they belong to. Health ambassadors were recruited for the project and were representatives of the study population. Throughout the project, we sought their constant input on the study design, topic guides, recruitment strategy, data collection process and feedback on the study findings and manuscripts. Based on their valuable feedback, we adjusted the workshop activities to be culturally appropriate, improved the clarity of our topic guides and refined our recruitment plan. Additionally, we sought informal feedback on the recruitment process from some of the participants during the early stage of data collection. One feedback was that community members might be reluctant to participate due to language barriers. We modified our advertising approach to highlight that two of our researchers spoke Punjabi, Urdu or Arabic languages and could provide support if needed.

### Participants and recruitment

A purposive sampling process targeted participants by their role, ethnicity and gender, aiming to recruit between 10 and 14 participants for the workshop, and aimed to have between 4 and 6 participants per FG. For the workshop, representatives of organisations within the Muslim community in Bristol, personnel who volunteer or work in local mosques, madrasas, Sunday Islamic schools, representatives of voluntary sector organisations that deliver health promotion and stakeholders from Bristol Council Public Health were recruited. For the FGs, parents of Muslim children who either attended madrasas or online Islamic classes were invited to participate. There was no pre-existing relationship between the research team and the participants prior to commencement of the study. However, some participants may have been familiar with the Health Ambassadors through their involvement in other community-based research projects or health initiatives. No interviewer characteristics, biases, assumptions or specific interests in the research topic were disclosed to participants. The study invitation materials stated: ‘We are researching this because childhood obesity in the UK is high. Mosques and Madrasahs could play an important role in supporting and encouraging healthy lifestyles among communities. We would like to see if this can happen in Bristol’.

Our health ambassadors advertised the study via email, in person or through community digital communication networks (WhatsApp) to the target communities and distributed the study’s invitation letter or poster, both of which included a link to a short demographic and contact details questionnaire hosted on an online survey platform^36^ (an online survey platform tool used for Academic Research and Public Sector organisations). The demographic questionnaire collected data on gender and ethnicity, as well as which stakeholder groups participants aligned with. For FGs, we also asked parents whether their child/children attended a madrasa or online Islamic classes. This information was used to check their eligibility for the study. Interested individuals who filled in the questionnaire were followed up by the research team via email or phone, provided with a participant information sheet and invited to sign a consent form. Written consent was obtained from each participant either in person or online by completing a consent form hosted on online surveys.

### Study procedures

Data collection was conducted between November 2024 and June 2025. The co-production workshop took place in November 2024 and was followed by an initial analysis to inform the design and the content of the FG discussions. Based on advice from the Health Ambassadors regarding participants’ availability during public, cultural and school holidays, the FGs were conducted between April 2025 and June 2025.

#### Workshop

The workshop was conducted face-to-face and lasted two and a half hours with a sample of n=15 participants (see participants’ demographics in the Results section). There were four participant tables, divided by gender (two tables per gender), with 3–4 participants per table, alongside 1–2 researchers (NAH, EC, RJ, JH-S, SEB, SAD) or health ambassadors (SB, ZH) to facilitate the conversation. Only participants and researchers were present; no non-participants attended the sessions. The workshop topic guide was developed iteratively by the research team, guided by the principles of co-production and the NPT. The topic guide was not pilot tested. However, it was reviewed by the health ambassadors for clarity and appropriateness. The workshop was split into four consecutive sections. Section 1 (30 min): knowledge share; short presentations on the research topic and the Bradford model. Section 2 (15 min): reflections from the participants on the programme. This section was guided by the coherence and the cognitive participation constructs of the NPT and aimed to explore the participants’ understanding of the programme and their level of engagement. Section 3 (35 min): understanding the context of madrasas in Bristol and exploring other physical activities programmes available in Bristol, guided by the coherent construct of the NPT. Section 4 (45 min), guided by the collective action and the reflexive monitoring of the NPT, aimed to collect insights on the programme transferability into Bristol and co-produce an implementation plan (online supplemental file 1). Following the workshop, a set of preliminary findings was prepared, emailed to all participants, and their feedback was invited.

#### Focus groups

FG discussions were conducted online and facilitated by researchers (NAH, EC) or health ambassadors (SB). Both EC and SB were trained by NAH in the facilitation and delivery of the FGs. A semi-structured topic guide was used to explore parents’ perceptions of their children’s health and PA, current PA and healthy diet programmes available in Bristol, the current madrasas’ context in Bristol and the transferability of Bradford’s programme into Bristol (online supplemental file 2). The topic guide was developed iteratively by the research team, guided by the principles of the NPT. The topic guide was not pilot tested. However, it was reviewed by the health ambassadors for clarity and appropriateness. Additionally, discussion points arising from each FG were incorporated into the topic guide to be explored in subsequent FGs. At the start of each FG, the researcher delivered a 10-minute presentation on the research topic to provide context. Participants were also shown a short video outlining the aims and the reach of the Bradford programme. The FGs lasted an average of 64±16 min, excluding time spent on introductions and video viewing. Due to last-minute participant withdrawals or insufficient enrolment for some sessions, FGs 4 and 5 were conducted with a single participant and therefore ran as individual interviews. FGs 1 and 3 each included two participants. Only participants and researchers were present; however, two participants’ children briefly entered and exited the session. Participants of the workshop and the FGs received £25/hour gift vouchers as a token of appreciation for their time and participation.

### Analysis

The workshop was audio-recorded, transcribed by a transcribing company approved by the University of Bristol and anonymised by the research team (EC). The FGs took place online using Microsoft Teams; therefore, Teams’ dictation software was used to transcribe the FGs. Accuracy checking of the transcripts and anonymisation were carried out by researcher EC, who listened to each recording and verified the transcripts’ accuracy. Participants were not invited to review or provide feedback on their transcripts. Transcripts were uploaded to NVivo qualitative data software to support data and codes organisation while conducting analysis. Data were analysed thematically using the guidelines described by Braun and Clarke.^37^ The thematic analysis process required following the procedures of familiarisation by anonymising, reading and re-reading the transcripts, coding, developing an analytical framework, charting and interpretation of the data. The analysis of each data set was conducted separately following the completion of each data collection component. For each data set, two researchers (NAH, EC) read all the transcripts and independently double-coded one transcript to assess the consistency of coding. Variations in coding were reviewed, discussed and a coding framework was developed inductively from the data (NAH, EC). The coding frameworks organised the individual codes hierarchically into broader domains representing barriers and enablers to the programme adoption in mosques and madrasa settings, which informed the final themes. The remaining transcripts were coded by one researcher (EC). On completion, the two researchers (NAH, EC) met and discussed the codes to review for further refinement. As the study adopted a multiple methods qualitative design, we (NAH, EC) were able to apply methodological triangulation within methods (the combination of two or more data collections, in this case qualitative methods, to examine the same phenomena).^38^ Following the co-production workshop, which brought together multiple stakeholders involved in madrasa delivery and health promotion for the target populations, we conducted (as described earlier) thematic analysis to identify codes and themes. This analysis informed the discussions of the subsequent FGs, allowing for a deeper understanding of the identified codes and themes. After completion of the FGs, a further thematic analysis was undertaken on the FGs data which generated additional codes and themes. The analyses from both data sets were then brought together, enabling the development of overarching themes.^39^ In this case, the two qualitative methods generated complementary data and strengthened the credibility of the findings through within-method triangulation. From the codes, themes and subthemes were identified and mapped into the four constructs of the NPT. Quotations from the workshop and FGs were selected and used to support the identified themes. We did not ask participants to provide feedback on the final study findings. However, the Health Ambassadors reviewed the findings and provided feedback, requesting clarification on certain points and the inclusion of additional illustrative quotes to strengthen interpretation.

### Positionality and reflexivity

NAH is a female senior researcher, PhD, with expertise in qualitative research. EC is a female field worker, MSc, with training and experience in qualitative research. SB (female) and ZH (male) are health ambassadors. RJ (male), JH-S (male), SEB (female) and SAD (male) are senior investigators with expertise in health research. During the analysis stages, both EC and NAH met regularly to discuss the analysis process, including reflexive discussions about how their own experiences might shape data interpretation. EC reflected on her experiences of PA opportunities during school years and the influence of the social networks and available opportunities on her choices, while NAH reflected on her experiences of cultural norms surrounding PA while growing up. RJ and JH-S are relatively new to conducting research with Muslim communities but have extensive expertise in PA research. SEB and SD had prior experience working closely with the Bradford research team and communities involved in developing the original intervention.

## Results

### Participants characteristics

#### Workshop

All organisations or Islamic settings invited to participate in the workshop (n=15) sent representatives, with the exception of two: one did not respond, while the other responded several months later, indicating that they had missed the invitation. Table 2 presents the characteristics of the workshop participants. Almost half of the participants were female; however, representatives from mosques/madrasas were predominantly males with only one female participant attending as a madrasa teacher. All participants were based in Bristol, apart from one representative of an Islamic organisation in Bradford who had experience of the Healthy Madrasa programme in Bradford.

**Table 2:** Workshop participants’ characteristics (n=15)

Characteristic	N
Gender	
Female	7
Male	8
Role	
Representative of Islamic organisation	3
Representative of mosques/madrasas	6
Representative of charity organisations	3
Madrasa teacher	1
Public health expert	2

#### Focus groups

35 individuals completed an expression of interest form, of these, 19 participants, all females, took part in eight FGs. Reasons for dropout included illness (n=2), work or family commitments (n=2), misunderstanding of the programme’s purpose (n=2) and unknown reasons (n=6). The characteristics of the FG participants are presented in table 3. All participants were female, with almost half identifying as being of Pakistani ethnicity. Participants reported that their children attended one or more than one Islamic religious school or had moved between different settings.

**Table 3:** Focus groups participants’ characteristics (n=19)

Characteristics	N	%
Gender		
Female	19	100
Ethnicity		
Pakistani	8	41
Indian	3	16
Bangladeshi	2	11
Black, Black British, Caribbean or African	3	16
Any other Asian background	2	11
Arab	1	5
Type of IRSs that their children attended		
Madrasas	13	48
Islamic schools (weekends)	7	26
Islamic courses online	7	26

IRS, Islamic religious school.

**Figure 2:**
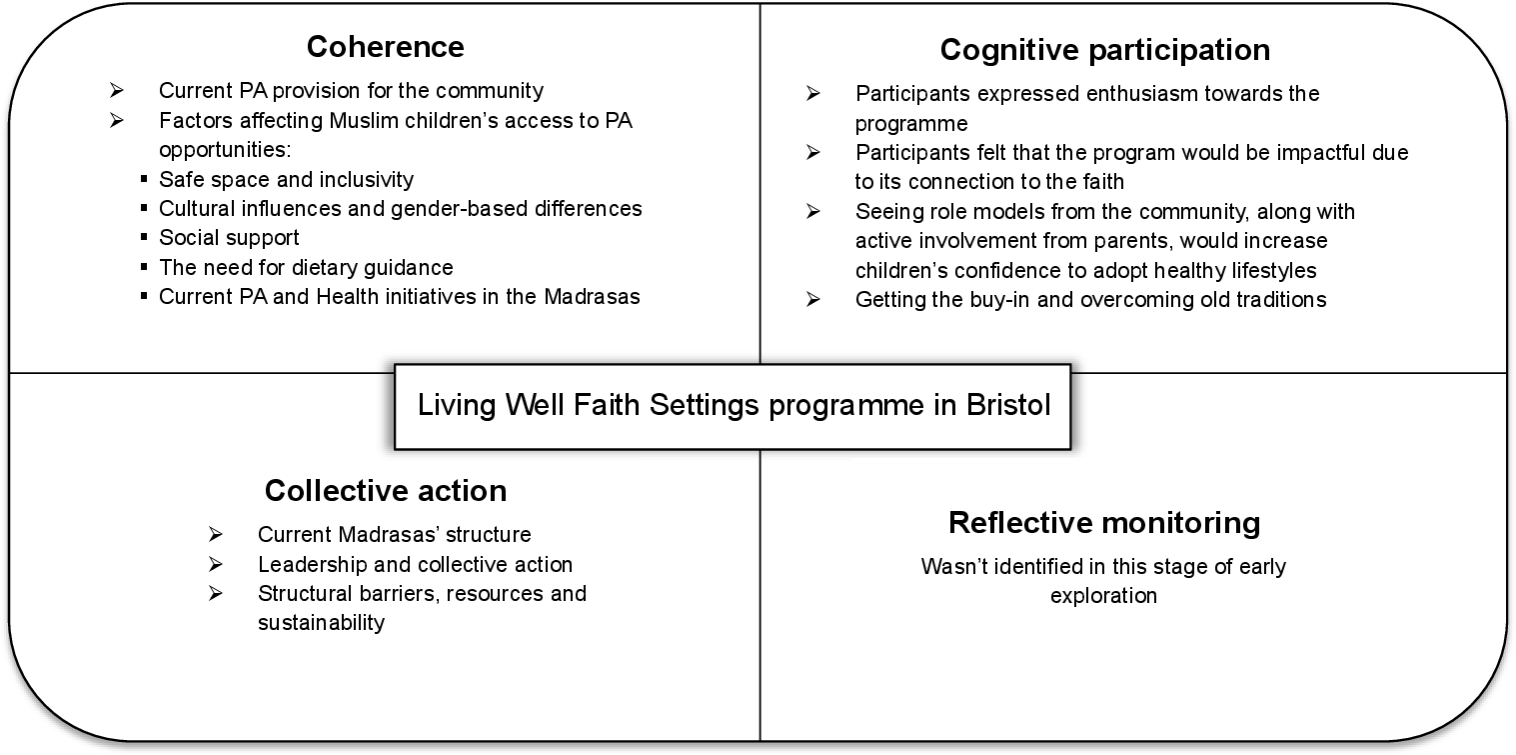
The workshop and the focus groups’ findings mapped to normalisation process theory. PA, physical activity.

Three main themes and thirteen subthemes were identified from the workshop and the FG discussions and mapped into three constructs of the NPT (figure 2).

### Theme 1: the need for PA and healthy eating programmes for Muslim children in Bristol ‘coherence, NPT’

#### Current PA provision for the community

Overall, participants stated that schools offered various opportunities for children to participate in PA, either during physical education lessons, at break times, or through extracurricular activities and after-school clubs. Other PA opportunities were offered through local leisure centres, community groups, or local mosques and madrasas. Community cricket clubs seemed to be popular among Muslim and South Asian children, with some clubs initially aimed at the Pakistani community. A parent also mentioned that the Somali community occasionally organised football games and barbecues in the summer for the community. While some parents were aware of available PA opportunities, others seemed less informed about what PA programmes, if any, were being offered within their community. Opinions were mixed on whether children were active enough or whether they enjoyed a healthy diet. Some parents felt their children were overweight or did not engage in enough PA. Others thought they had noticed children’s activity levels declining throughout school as they grew older. On the other hand, several parents found that their children were very active and engaged in various sports without any concerns for their health.

Uh, it’s a lovely school, um … lots of, lots of, uh, ethnicities, a lot of nationalities as well. But you could clearly see high levels of obesity amongst children, and sometimes I notice that it’s more pronounced amongst the girls than the boys. (P1, FG6)

I think age has a big factor, so when they’re in primary school, […] they run around anyway […]. My younger boys always … they are very active, and my older two used to be like that, but as soon as they hit secondary school. It just completely changes […]. My daughter used to be really active, and now she just … she just isn’t. (P2, FG6)

#### Factors affecting Muslim children’s access to PA opportunities

Many participants identified several factors that influence their children’s access to PA opportunities, as well as their attitudes towards exercise and diet, these included the following sections.

##### Safe space and inclusivity

Concerns around inclusivity and safety for Muslim children in PA sessions were raised by some participants. Some parents who lived in areas where Muslims were a minority reported on instances where they have felt uncomfortable sending their children to sports clubs out of concerns of their children being subject to social isolation, bullying or worries of loss of cultural identity. Some parents instead preferred, if they had access, to send their children to clubs that are aimed at specific groups, such as the Pakistani cricket club.

Yeah, he’s saying I want to go on weekends. He’s saying and yeah. And even I’m looking now for him any groups where he can go. I found few groups like nearby. But I heard that there’s just all white people. I’m just a bit scared as well to send him in them. I don’t know it’s just me or but I heard that there’s some people bully or neglect the child who’s come from like Asian background, especially Pakistani, Muslim. So that’s why I said no, no for them. He brings some leaflets home. He said they’re running the groups and then I search up and say, no, I’m scared. (P1, FG1)

But I take the kids to [a] running club, […] two more families that are Muslim families, everyone else is, um … is white ethnicity […]. So … And there was a recent event at the club where, you know, boys, and they sent each other pictures that were not good, and one of … one of the recipients of those nasty pictures was from the Muslim family, and the mother found that very offensive, and then all of a sudden, the club became sort of a hostile environment for her child, and so although it’s sort of, it seems harmless, but she and me and her had a conversation in that she’s thinking about withdrawing her son, and her son has been with that club for years now, but she was worried that if she withdraw him from the club, he will have … he will put on weight, because he’s taking steroids as well for his health conditions. But … you know, it’s … the club did nothing, to be honest. […] all of a sudden, that became at risk of being inaccessible to a family that really, really understands the value of sports. (P1, FG6)

##### Cultural influences and gender-based differences

There appeared to be a difference in opportunities and attitudes towards PA for Muslim girls versus boys. PA opportunities for boys, such as cricket and football, were popular in the community. On the other hand, culturally accepted PA opportunities for girls were less common. Some parents expressed how some girls were reluctant to take part in sporting clubs in schools due to PE being mixed. They also felt that covering up may also hinder their abilities to fully take part sometimes, especially when doing PA with boys.

To walk to and from school. I think … I mean, obviously, she’s a girl, she covers, she’s not really gonna be that comfortable running around in public. She does school PE, but she doesn’t do it to the best of her ability, because she’s in mixed classes, and she doesn’t want that attention. (P2, FG6)

I think one of the other barriers is as you get older. You may want gender only activities. Which I know is really lacking. (P4, FG8)

The concept of mixed gender PA initiatives, and the feelings of safety and discomfort also appeared to be a barrier among the adults’ PA levels, and they discussed the importance of having a safe space for Muslim women and girls to partake in PA.

Also, I enjoy playing netball. So I actually went on, this [website name] website. And I found a coach that was doing Netball and I just messaged her, and I asked her if it’s women only, and if it’s overlooked by men, and she said, it can be completely private. So I take one of my daughters to that, and we play together. So that’s really nice, and my coach is lovely. She always tries to make it accessible to Muslim women who are interested in joining. (P3, FG8)

Different PA initiatives hosted by the mosque or madrasa also appeared to be readily available to boys. Some also expressed a mindset that some activities were more suitable for teenage girls such as cooking or painting.

So, in Bristol, I can’t find things for her to do. She would like to do things. She does, you know, she plays basketball, netball, and other activities, you know, after school with her school. However, um, you know, like my son has attended things with, um, our local masjid, like, they’ve organized during the summer holidays, you know, like, they’ll encourage the children to come and read Salah, um, you know, and then followed by playing football and things, but there’s nothing like that for the girls. (P1, FG7)

No, no, they were teenagers, the girls. So I used to take them to another cooking club, something like that, something related with her age. (P2, FG7)

##### Environmental factors

Access to green spaces was discussed. While some parents report living near green spaces, having convenient access to parks, others discussed living in areas of high-rise buildings with limited suitable spaces to engage in PA. Other factors included parents having limited ability and time to take their children to and from various activities, madrasas and clubs, especially if they had multiple children. Costs and affordability of after-school clubs were also a common barrier.

In terms of after-school clubs, again, money’s gonna be a big thing, especially for people with big families, and you’ve got multiple children, one is access in terms of taking them to and from to places is an issue […], and the mental stress of managing it all … Is an issue. (P2, FG6)

So for me, it’s like distance, it’s cost-wise, it’s location that stops me from helping him in wanting to be active (P2, FG1)

##### Social support

Participants felt that parents were responsible for getting their children involved in PA, and how much they prioritised PA had an impact on their children’s level of PA.

I think it is down to the parents. So some parents are generally more active or want to become more active, and some parents just not used to that lifestyle. […] But then you get parents who have not had that experience and want their children to experience it, and they push their children. (P2, FG8)

There also seemed to be more fathers and sons’ (and sometimes daughters) activities than mum-led activities. Gender roles, caring responsibilities and lack of opportunities massively hindered mums’ abilities to prioritise PA for themselves with their kids.

And recently, my husband and my son start cycling on Saturday morning […], It is a gap because maybe traditions or culturally moms are very busy. They’ve got more responsibilities. House responsibilities So the men, they go, they say we’ve got earning responsibilities we providing everything, but still they’ve got the time they can do their physical activities but moms, we’ve got household responsibilities, but they can’t find the time to do these things maybe i don’t know. (P1, FG1)

how do we ensure that we are kind of doing things as a family, as opposed to just the mum, and then she has to worry about what she’s got to do with the kids. I don’t know whether even the mosque has capacity to be able to put … do … deliver things like that for example, to pick them up and can it be mother and daughters, and if they’ve got little boys, can they also bring them along? (Female, Representative of charity organisation (workshop))

Friends and the larger community appeared to affect children’s and parents’ involvement in PA. Parents often noted that their children were more likely to try a sport or club if their friends were doing so, and vice versa.

You know, parents are giving importance to this, and it made me give importance as well you know, looking at the other parents. (P3, FG6)

### Need for dietary guidance

Some participants noted that food was an important part of their culture and that making changes to dietary traditions while managing complex power dynamics (eg, elders’ and men’s food preferences) could be challenging. On the other hand, some parents discussed how cooking meals from scratch and avoiding processed food was also a typical characteristic of their culture’s dietary approach. Some parents also actively prioritised feeding their children a balanced diet.

So, diet is very, very, very important, like, very important as a parameter here to consider, because in our communities, you know, diet and food and getting the family together, is crucial to our social interactions and how we do things. But then, also, it makes it hard sometimes if you have, there is generations in one household … To change anything, and you’re cooking for … according to the preferences of older, maybe, or … the men vs the women, or, you know, it makes it harder to make changes. (P1, FG6)

Parents also discussed trying to navigate healthier ways of snacking, as some children were habitual snackers, opting for market-available, unhealthier options high in sugar. Meanwhile, some parents mentioned that they had made a conscious effort to encourage healthier snacking habits, such as eating more fruits instead.

Um, but I think the big … the big challenge … I find for myself, and I see for a lot of families, is the snacks. Um, finding healthy snacks is quite tricky. (P1, FG4)

Parents felt that if dietary guidance was offered, it often was not tailored to their culture, and therefore, it was difficult to follow and to make healthy changes to their family’s diet. They expressed that they wanted access to culturally tailored dietary guidance and education, which would make it easier to approach making healthier choices at mealtimes.

And also giving advice that was suitable to … the South Asian communities, as opposed to, you know, you go to the doctors and they’re saying, oh, why don’t you eat more asparagus and more salmon, and you know, this and that. And that’s great, but how many people realistically will go home and start cooking asparagus? As Asians, that is not something that we do. (P1, FG3)

In relation to diet and culture, workshop participants discussed traditional Islamic cultural norms around body standards and perceptions around ‘healthiness’, and how they might not align with current recommendations around diet and exercise.

But initially er back to the parents, the elders, a lot of them back in the days, you know, begin big was attractive, you know, especially when it comes to our mothers, like a lot of them, they used to, the fathers used to think that the bigger you are the more beautiful you are, you know, that was that culture. (Male, Representatives of mosque/madrasa (workshop))

### Theme 2: The current context of madrasas in Bristol ‘coherence, collective action, NPT’

#### Current madrasas’ structure

Participants explained that madrasas in Bristol typically were held either on some weekdays, following school hours (1–2 hours) or on weekends (2–4 hours). Online Islamic teaching became a preferred option for some participants following the COVID-19 pandemic, due to its convenience. Many parents valued the social benefits that the madrasas offered, as they provided their children with the opportunity to meet and build friendships with children from the same community, thereby learning Islamic ethics while maintaining social connections, cultural identity and links.

Yeah, but madrasa is good, because they can communicate with other children. They’re sharing their feelings, they, uh, they have activities as well. Love, laughter, you know, emotion. And you make lifelong friends as well. Sharing, so … Yes, that’s true, but it’s not convenient in every city, that’s the problem. (P1, FG2)

I would say it’s the way to establish our identity as Muslims. It’s the only way we can do it living in the West. It’s via the mosque you know that’s our standing pinpoint. And then through that we teach our children about Islam. For me, it’s identity shaping as much as learning as well. (P2, FG1)

However, some parents felt that some madrasas can be intimidating, as their teaching styles were conservative or strict, which although it might be an effective learning environment for some, may not be so effective for others, and can be off-putting for children. Some parents also mentioned that certain madrasas may be influenced by specific cultural perspectives, making them less inclusive or relatable for children from other backgrounds.

At first, he was going to a madrasa in a mosque. Um, and he didn’t really get on … it was a very old-school style of … very … lots of children expected to just sit and read the alphabet. […]. So, we took him out, […]. So now, he does [go to a weekend madrasa with a different teaching style and activities], but he also does the Quran. Uh, one-to-one lessons online. (P1, FG4)

#### Current PA and health initiatives in the madrasas

Participants recalled that some madrasas often ran some form of activities during the summertime, such as football or cricket competitions. Madrasas with longer attending hours or those that ran at weekends were reported to encourage children to play or do PA during break times. On some occasions, some madrasas hired out community or school halls to host activities, as many madrasas lacked the physical space to run such activities. Some also discussed how some madrasas offered children healthy snacks, such as fruits. However, some parents report that they were not aware of any current health-focused initiatives offered by the madrasas, but that mosques did sometimes host health-oriented talks.

Basically [a weekend Madrasa with longer attending hours] have Islamic studies. Um, but then within that time, they have a 45-minute session of sports. Um, and in that sports session, uh, the younger years are, you know, they’re given different activities for them to take part in, […] like, games and things like that set up, so they’ll do that, but as they get older, […] You know, like, the kids can actually play it, like, for example, the boys will choose whichever sports they want to do, but it will be, like, basketball. You know, football. Rounders and things like that … (P1, FG7)

Those sessions which are around 2 h long and in the middle the children have breaks, and in those breaks they actually have fruit […] It goes down really well. And also that bit of running around depending on what age group it is. (P4. FG8)

But, um, the mosques here, I mean, Bristol they are trying to sort of engage the youth a lot more and they are doing activities where they’re doing. We have the inter, inter mosque football tournaments. But we still—but what we all did agree with is that it is still very male oriented where the girls there is lack of provision for them. Our mosque in [town] is fantastic, the work that they’re doing with this, but I think the girls, there’s not very much for them. (Female, Representative of Islamic organisation (workshop))

### Theme 3: Adapting the Bradford programme into the Bristol context ‘cognitive participation, collective action, NPT’

Many participants expressed enthusiasm towards the Bradford Healthy Madrasas programme. Participants felt that the programme would be powerful in impacting the behaviours of children from the Muslim community around PA and healthy living due to it being connected to the faith. Some participants also noted that seeing PA champions from the community, along with active involvement from parents, would increase children’s confidence to adopt these practices even outside faith settings. They also suggested that the programme would be beneficial not only for physical well-being but also for mental well-being.

Amazing, uh, initiative, much needed for all the reasons that we discussed, […]. Um, and they are actually … the faith settings are quite powerful in influencing behaviour, because, um, normalising that activity in a faith setting, you know, sends a message to parents that this is something important to do, and something that should be prioritised. (P1, FG6)

We need to kind of push it from the younger years. So that it’s kind of normal for them (P1, FG7)

In terms of practicalities and feasibility, participants felt that the programme could be replicated, but they also pointed out similarities and differences between Bradford and Bristol that would need to be considered to ensure effective implementation.

#### Differences between Bradford and Bristol

Participants highlighted two key points, of which the Muslim community in Bristol differed from that in Bradford. First, Bradford has a larger Muslim population which may offer access to a larger pool of volunteers and established services. Second, the Muslim community in Bristol is more ethnically diverse and geographically dispersed, despite a significant concentration of residents in the inner city and the northeast region of the city. Participants pointed out that these distinctions should be carefully considered when adapting the programme, particularly in relation to gaining community buy-in, forming an appropriate leadership team and securing resources.

And, um, you know, so sort of a … maybe in Bradford, there’s a huge … there’s a bigger community, ethnic minority community, and faith and Islamic community. Whereas here in Bristol, it may be that it is sort of a bit segregated. It’s not all … it’s not that big, and it’s not all focused in one location. So, we may need to consider that, um. […]. Some mosques are more versatile, and you get multiple ethnicities going in, but others may be more focused on one. (P1, FG6)

The difference between here and Bradford, they’re well more … much more established, especially when it comes to weekdays so madrasas, their syllabuses are much more organised; number of students, number of teachers, and how the communities are on the whole, er that’s much more connected, a lot more people there […] Here, from my experience anyway, I must state; it’s not as well organised; they’re short on people, short on staff, short on volunteers. (Male, Representatives of mosques/madrasas, (workshop))

#### Getting the buy-in, overcoming old traditions, awareness and communication

Participants emphasised the importance of securing engagement across three levels: community leaders, Islamic teachers, parents and the wider community. Overall, the message to get the buy-in needs to be culturally appropriate and tailored to the needs of ethnically diverse communities. Without this, there is a risk that some communities may engage more fully than others, potentially undermining the programme’s inclusivity and overall impact.

The engagement with the … with the … with the faith settings needs to be diverse, and maybe the messaging, or the way you engage with the faith settings may differ if you’re engaging with Bristol Inner City, or Outside of that. (P1, FG6)

P1: I feel in Bristol we’ve got a little bit of an issue that our among our Muslim community people who are, for example, Arab want to do something just with Arab. Pakistanis want to do something just with Pakistanis, Somalis same thing […]

P4: you are right, like, for example, scouts mainly it’s South Asian children that turn up and only a couple of children from the Somali background, […], all the volunteers are South Asian, so it sometimes ends up being, if you’re not a multicultural, multiethnic group of volunteers working together. It also has that impact. (P1, P4, FG8)

Some participants recognised that some mosques and madrasas (as well as in the broader community) may hold established cultural traditions, which could present challenges when adapting the programme, and that they carried an element of ‘conservatism’. This is particularly around children running around in mosques and promoting PA activities for girls.

Egos, like with everything. I think, like, with everything, it would be egos that get in the way. As well, you know, sort of religious conservatism. ‘Well, we never did it like this, our children learnt just fine’. ‘You know! where’s the need to … be jumping up and down? If they’re at madrasa, they should be sat down’, you know, that sort of idea. (P1, FG4)

Female participants vocalised that they felt they were not listened to as much as the men and that their opinions and initiatives often got lost. They emphasised that there is a problem in communications and stressed the particular importance of making mothers feel welcome, bringing their children into the mosque and providing equal opportunities for women. Those participants stated that this conservatism stemmed from old cultural traditions, whereas Islamic guidance provided many examples that supported using mosques as community hubs and promoting meaningful participation from women.

[…] the men take the lead. In Bristol I’ve been to committee meetings. We are trying to set up this thing where a lot more people get involved with the community. It’s the men in there … very different. And it was like everything we suggested, oh yeah. It’s a brilliant idea. But, um, no, my idea’s better. It is like we don’t—our opinions don’t count. (Female, Representative of Islamic organisation (workshop))

It can be, as you said, the brothers are saying there’s things going, the sisters are saying there aren’t. Now, in between is your communication problem, […], what I’m saying is be aware that the other half of the population’s not actually getting that message that the other half thinks they’re telling and it’s not, or not listening either. (Female, Representative of charity organisation (workshop))

The Imams and Islamic scholars acknowledged the gap. They discussed how local mosques were already making conscious efforts to encourage more women’s participation and recognised that it was something that could be further improved.

We realised that it was important that whatever is being offered to the boys, to the men, is equally offered to the females as well. (Male, Representatives of mosques/madrasas (workshop))

Participants emphasised that raising awareness and ensuring effective communication were essential to overcoming old traditions and securing the necessary engagement from different groups. Islamic scholars and Imams spoke about the need to run regular workshops focused on healthy diet and PA, to address the lack of knowledge within the community and to help foster a deeper understanding of the need for the programme.

We need a lot more awareness in Bristol to educate our community um as well as more workshops related to obesity, things like that. (Male, Representatives of mosques/madrasas (workshop))

And in reality, it is about awareness, educating madrasas or mosques, and helping the imams play, play a righting role in that. But communities need to have awareness, parents need to handle that. It’s not a one person’s responsibility. (Male, Representatives of mosques/madrasas (workshop))

#### Leadership and collective action

Workshop participants suggested that the first step in implementing the Healthy Madrasas programme would be to form a new committee with a neutral setup. They agreed that establishing an ‘Islamic community-led’ *steering group* would be more effective than relying on an existing set-up, particularly in facilitating an inclusive delivery of the programme. FG participants also observed that Bristol’s faith settings were segregated, and that there was a need to work collaboratively and define roles and responsibilities in programme delivery. Additionally, building effective partnerships with other relevant organisations was also highlighted as key in ensuring the programme’s successful delivery, including charity organisations, the council and schools.

You need a neutral setup so it can encompass all these, […], because at the moment we’re fragmented. (Male, Representatives of mosques/madrasas (workshop))

I think that mapping of what are the community organisations that are willing to deliver this in faith-based settings, but most importantly have the understanding of the needs and prejudices that people go through, you know, and that communities are impacted by. (P1, FG6)

#### Structural barriers, resources and sustainability

Many participants identified costs and funding as significant barriers to a successful implementation. Some mosques lacked enough space, equipment and experience in securing financial support. Another concern raised by the participants was a lack of trust surrounding funding sources. There was apprehension about whether financial support might come with conditions that conflict with Islamic values and beliefs, such as funds obtained through lottery schemes. Further concerns were also raised in relation to the sustainability of the programme, as participants noted that previous initiatives, such as sports clubs, had struggled to remain open due to a lack of funding. Some workshop participants suggested that partnering with other local mosques, madrasas, schools or community centres to use existing resources and facilities should be encouraged.

everyone’s always crying about funding and not being able to fund it. I think that is the biggest thing (P1, FG7)

And every single time, people talk about an initiative that had a lot of impact, um. It gets stopped for some reason, the funding stops. (P1, FG3)

Or sometimes also where that money is coming from, it could well be that lottery, […], and obviously it’s not Islamically acceptable to take money as such, whether it comes from erm … [lottery], […] and then just to add the second part, strings attached, is there any strings attached to it? (Male, Representatives of mosques/madrasas (workshop))

Finally, participants emphasised that responsibility should be taken by the community to deliver the Bradford programme in Bristol successfully, and that the responsibility should not be solely on Imams and Islamic teachers. This need was in the shape of having the willingness to give up some time to volunteer and to engage with the programme. Parents suggested that Bristol’s Muslim community had already demonstrated initiative in volunteering, and some expressed that the community could be further encouraged to do more if it meant benefiting the children of the community.

It’s really interesting to see Because, and again, I keep bringing it back to [Islamic faith setting running an initiative]. Because I do think that it’s … it’s … it’s outstanding in what it’s doing. For Bristol, in that … The number of volunteers, the number of parents who are there who volunteer. […]. Is … it’s really heartwarming to see. (P1, FG4)

## Discussion

We aimed to investigate the feasibility of adapting a childhood obesity prevention intervention, originally developed for a UK city (Bradford) with a high proportion of Muslim communities, for implementation in a different UK city (Bristol) with a lower proportion of people from the Muslim faith. Our findings present an early phase exploration of stakeholder perceptions on the programme and on its transferability. Our findings suggest that in cities where Muslims constitute a smaller minority than Bradford, existing PA provision might not ensure equitable access to PA for Muslim children. This is due to societal and cultural barriers that current PA programmes often overlook, such as ensuring a safe and inclusive environment for all, and the need for culturally acceptable PA opportunities for Muslim girls. In terms of the transferability of the intervention, our findings identified two key points that require attention: the steering committee of the programme needs to be neutral and inclusive of culturally diverse communities, and Bristol Islamic religious settings were perceived to have fewer resources, including manpower, financial resources and organisational structure.

Our study indicates that girls from the Muslim community may face compounded barriers to access mainstream PA opportunities, due to cultural and religious norms, concerns around safety and inclusion, and limited availability of culturally appropriate PA activities. Many studies have shown that girls are often less active than boys,^40 41^ and although PA levels tend to decline with age, this decline appears to occur earlier among girls than boys.^42^ However, it was demonstrated that levels of PA among British Pakistani girls were lower than those observed in white British girls, including in key active behaviours such as organised sports and exercise, outdoor play and active travel to school.^43^ While barriers such as perceptions of body image, lack of comfortable and suitable sport clothes, and limited enjoyment of available PA activities affect girls’ access to PA (including Muslim girls),^44^,^47^ religious and cultural norms are frequently cited in the literature as specific and significant barriers for Muslim girls and women to participate in PA.^48 49^ These barriers appear to be more of an issue for teenage Muslim girls and Muslim women. However, for younger girls, parental and family support and concerns about neighbourhood safety were found to be contributing (or hindering) factors to participation in PA^43 50^ (also identified in our study). Interestingly, despite these barriers, the female participants in our study were very keen to find culturally acceptable PA opportunities, such as girls or women-only PA sessions. However, they highlighted the paucity of such opportunities in their community. Recent research commissioned by the Muslimah Sports Association and conducted by Muslim Census in 2022 supports these findings.^51^ Their online survey of 319 Muslim females aged 18–45 years explored motivations and barriers to PA participation. They found that 97% of women surveyed wanted to increase their current participation in sports, 80% stated they would be likely to attend women’s only sport sessions if they were available, however, 65% answered ‘no’ when asked whether they were aware of any women’s only events or sports associations who run appropriate activities. The lack of women only spaces/facilities was highlighted as a predominant barrier that appeared to prevent participation in sports. Taken together, these findings suggest that there is a critical need to move away from a sole focus on social and cultural norms that hinder PA participation. Instead, efforts should prioritise removing barriers embedded in structural inequalities that Muslim women live with and on the development of practical, inclusive, and culturally responsive PA opportunities that meet the needs of Muslim girls and women.^52^

Difficulties and mistrust in accessing mainstream PA opportunities were also highlighted in our study as factors affecting Muslim boys’ participation, particularly in areas where Muslims constitute a minority. Parents expressed concerns about their children being subjected to social isolation, bullying, racism or loss of cultural identity, which often led them to deny their children’s wishes to engage in PA. Reports of Islamophobia and racism in sports have been frequently featured in the media. A recent study by Awan and Zempi examined the qualitative experiences of 28 male and 12 female Muslim football players at the grassroots level.^53^ The findings revealed that players experienced Islamophobic incidents both offline and online, perpetrated by fans and other players. Female players who wore the hijab reported facing a ‘triple penalty’ of Islamophobia, racism and misogyny. The study highlighted that as a result, the participants did not feel they could encourage other Muslims to engage with this sport. Similar findings were reported by the Muslimah Sports Association, where 33% of respondents stated that past experiences had negatively impacted their participation in sports.^51^ Discrimination was also reported to be widespread in cricket.^54 55^ It was found that while 50% of all respondents had experienced discrimination in the past 5 years, the figures were significantly higher among ethnic minority players: 87% of those with Pakistani and Bangladeshi heritage, 82% with Indian heritage and 75% of Black respondents. Although most of these reports focused on organised sports, it is unsurprising that Muslim parents may feel reluctant to encourage their children to participate in extracurricular PA programmes, especially in less diverse areas.^56^

The Bradford programme has a unique potential to address this equity gap by providing PA opportunities that are culturally appropriate to the needs of the Muslim children as well as providing guidance on culturally tailored healthy diets and dietary habits. The theory of ‘Expanded, Extended and Enhanced Opportunities (TEO)’ posits that the primary mechanisms of change in youth PA interventions should fall into three categories: (a) the inclusion of new opportunities for youth to be active (Expand), (b) increasing the amount of time allocated for those opportunities (Extend) and/or (c) improving the quality of the opportunities (Enhance).^57^ The Bradford programme aligns within the ‘Expand’ category of the TEO framework, but it also incorporates a socio-ecological approach that addresses the complex interplay of the individual, interpersonal, organisational, community and societal factors influencing physical activity and healthy living among Muslim children.^26^ Uniquely, it embeds the notion that being physically active and healthy is part of Islamic identity. Participants in this study expressed views similar to those identified in Bradford (and Birmingham: another major UK city) during the programme’s early development phase.^16^ Additionally, the co-production approach followed in this study supported identifying a mutually agreed list of barriers and recommendations to consider for future adoption (table 4). This provides assurance that the barriers and the facilitators in the current study setting are of a similar nature to those faced in Bradford, suggesting potential for successful translation to the current context. Another caveat arises from an opinion expressed by one of our study participants that the programme would take the pressure away from parents to find suitable PA opportunities for their children. While this was a single viewpoint, the developers of the programme should ensure that the programme does not become the only acceptable ‘Islamic’ healthy living and PA opportunity within the Muslim communities and that it does not replace (but add to) the current mainstream childhood PA programmes. Similar trends have been observed in other PA interventions where pupils switched between PA clubs rather than increasing their overall PA levels, thereby weakening intervention effects.^58 59^ To address this, participants from both studies emphasised the importance of madrasas forming partnerships with other relevant PA organisations, schools and clubs. This would help position the programme as part of a collective societal effort to address the equity gap.

**Table 4:** Barriers and recommendations for implementing the Healthy Madrasas Programme in Bristol

Key area/theme/NPT domain	Identified barriers	Co-produced recommendations
Leadership and governance NPT: collective action	Muslim populations in Bristol are more ethnically diverse than Bradford and more geographically dispersed	Establish a neutral, Islamic community-led steering group Ensure steering group reflects ethnic diversity Build formal partnership between mosques, madrasas and schools, community centres, relevant charities and local authorities
Resources and sustainability NPT: collective action and cognitive participation	Limited space, equipment and funding; mistrust of funding sources perceived to conflict with Islamic values; concerns about volunteer fatigue and sustainability	Promote resource-sharing access settings and partnerships Secure culturally acceptable funding Develop support mechanisms for volunteers
Community awareness and buy-in NPT: coherence and cognitive participation	Limited awareness of healthy diet and PA benefits; entrenched traditions potentially limiting engagement	Securing engagement across three levels: community leaders, Islamic teachers, parents and the wider community Deliver regular workshops within mosques focused on healthy diet and physical activity to raise awareness The message to get the buy-in needs to be culturally appropriate and tailored to the needs of ethnically diverse communities
Cultural norms and gendered expectations NPT: coherence	Cultural and religious conservatism limiting girls’ participation; preference for gender-stereotyped activities	Frame PA within Islamic principles; promote faith-aligned message supporting girls’ participation
Cultural norms and the use of mosques and madrasas for PA NPT: coherence	Concerns about children running around in mosques; mothers feeling unwelcomed in mosques settings; some madrasas environment perceived as overly strict	Promote understanding of mosques as community hubs Encourage inclusivity and child-friendly approaches
Gender power dynamics and communications NPT: collective actions	Women’s voices and initiatives marginalised; ineffective communication between men and women committees	Improve communications channels targeting women Ensure women representation in leadership and delivery roles

NPT, normalisation process theory; PA, physical activity.

### Strengths and limitations

The main strength of this study lies in the support provided by the health ambassadors throughout the study including reaching the communities, helping to build trust, facilitating recruitment and data collection as well as providing feedback on the research findings. Their involvement in our project was vital for building trust and relationships with the communities we worked with, thereby enhancing the relevance and credibility of our research findings.

This study has several limitations. First, although the workshop was well attended by both men and women, no male participants took part in the FGs, despite offering various time slots to accommodate different working patterns. The absence of male caregivers in the FGs limits the range of the parental perspectives captured and may restrict the comprehensiveness of the findings. However, this outcome may be due to existing traditional gender roles and cultural norms. While the workshop was aimed at representatives of Islamic organisations, roles often held by men, such as Imams, the FGs were aimed at parents. Given that the caring responsibilities traditionally fall to women, it is unsurprising that all our parent participants were female. In this sense, the composition of the FGs is not only a methodological limitation but also reflects the social realities shaping parental engagement. Future research should therefore employ targeted and gender-sensitive engagement approaches to support the inclusion of male caregivers. For example, the Bradford study successfully recruited parents of both genders, likely due to the presence of a male researcher. The second limitation was the low participation from the Somali community. This may be due to not having as established connections with the Somali community. Additionally, due to recruitment challenges within hard-to-reach communities, several FGs were under-subscribed and ran with one or two participants (with single-participant sessions conducted as individual interviews). In these occasions, cancelling these sessions may risk excluding already recruited participants and would further exacerbate barriers to engagement. We therefore prioritised inclusivity and accessibility by proceeding with the scheduled sessions. Nevertheless, these sessions generated rich, in-depth accounts aligned with the study aims. We recommend that future studies in similar contexts employ hybrid formats to balance methodological ideals with feasibility.

## Conclusions

There is potential for the ‘Healthy Madrasas’ programme, developed and implemented in Bradford, to be adapted and delivered to other UK cities where the proportion of people from the Muslim faith is lower and the ethnic composition is more diverse. The programme would provide equitable access to PA for Muslim children, while current PA provision inherits societal and cultural barriers that hinder PA participation, such as ensuring a safe and inclusive environment, and the need for culturally acceptable PA opportunities for Muslim girls. In terms of the transferability of the programme, two key points emerged: the steering committee of the programme needs to be neutral and inclusive of culturally diverse communities, and Bristol Islamic religious settings were perceived to have fewer resources, including limited manpower, financial resources and organisational structure. Future work should pilot the programme in such settings to gain more insights into the implementation requirements.

## Supplementary material

10.1136/bmjopen-2025-115161 online supplemental file 1

10.1136/bmjopen-2025-115161 online supplemental file 2

## Data Availability

Data are available upon reasonable request.
