# Supplementary material for: Healthy Madrasas: a qualitative study using normalisation process theory and co-production approach to explore translation of a childhood obesity prevention intervention in Islamic religious settings from one UK city to another
Source: BMJ Open. 2026 Jun 29;16(6):e115161. doi: 10.1136/bmjopen-2025-115161 (PMC13331203; doi:10.1136/bmjopen-2025-115161)
Supplement: online supplemental file 1 [file bmjopen-16-6-s001.docx]

**Session 1: presentations on Bradford’s model of Healthy Madrasas**

**Session 2: initial thoughts on the program (15 min)**

All group discussions

Main question

- What are your initial thoughts on the programme?
- Any questions about the presentations?

**Session 3: How the context in Bristol differs from Bradford (35 min)**

**The context (circumstances) in Bristol**

| **Children’s health and physical activity in Bristol** | **How Madrasahs operate in Bristol** | **Other relevant circumstances** |
| --- | --- | --- |
| **Prompts for facilitators:** encourage the discussion around:   1. How **active** do you think the children in your community are? What factors do you think influence this behaviour?   What about their **diet**? What factors do you think influence Children’s diet?   1. What current or previous physical activity and healthy diet programs delivered outside of the Madrasa setting have benefited children from the Muslim community in Bristol? How would you describe what they did and what the outcomes were? Have these programmes linked up with Madrasas at all before? What worked or didn’t work about this, or if they haven’t, are there any reasons for this? [please note that some mosques run physical activity programmes like football competitions among] mosques] | **Prompts for facilitators:**  encourage the discussion around:  How many Madrasas are there in Bristol, and what are their denominations? How many children, or what proportion of children from the Muslim community, attend Madrasahs in Bristol? How many days do they attend—weekdays or weekend days? By ethnicity? Depends on where they live? Do many attend online lessons? What is the typical age range? Are there any challenges that Madrasahs face in delivering their work??   1. What are the views on mosques delivering health promotion campaigns? How are they finding working with other partners (e.g., NHS, community organisations)? Do the mosques in Bristol have any experiences or appetite for delivering health campaigns? How do they describe the response and engagement of the Muslim community with health campaigns delivered through mosques? | **Prompts for facilitators:**  Are there any other relevant circumstances we need to be aware of? |

**Session 4: The program transferability into Bristol (45 min)**

**The input in Bristol** (what is needed in Bristol to deliver this program)

| Steering Group | HM Local delivery organisation/Community engagement manager | Health group within each Madrasahs | Funding, skills and resources | Other |
| --- | --- | --- | --- | --- |
| **Prompts for facilitators:**  **Who** would be the steering group in Bristol?  Any **potential barriers** when establishing a steering group in Bristol?  How to **overcome** them?    **What activities** would the steering group facilitate? | **Prompts for facilitators:**  **Who** would be the delivery organisation in Bristol?  Any **potential barriers** when establishing a delivery organisation in Bristol?  How to **overcome** them?  How would the delivery organisation **engage the mosques?**  **What activities** would the Local delivery organisation/Community engagement manager do? | **Prompts for facilitators:**  **Who** would make the health group in the mosques?  Any **potential barriers** when establishing a Health group?  How to **overcome** them?  How would the health groups **engage the community**?  Would the health group be able to facilitate 9 toolkit activities over a 2-year period? | **Prompts for facilitators:**  encourage the discussion around:  1) What assets are available in Bristol to support this programme?  What funding is required? (How much would each setting need?)    What **training** is needed?  What other resources? | Any other aspects needed? |
