# Supplementary material for: Healthy Madrasas: a qualitative study using normalisation process theory and co-production approach to explore translation of a childhood obesity prevention intervention in Islamic religious settings from one UK city to another
Source: BMJ Open. 2026 Jun 29;16(6):e115161. doi: 10.1136/bmjopen-2025-115161 (PMC13331203; doi:10.1136/bmjopen-2025-115161)
Supplement: online supplemental file 2 [file bmjopen-16-6-s002.docx]

**Active Madrasahs: healthy eating and physical activity for children attending Islamic faith settings**

**Discussion guide for focus groups**

**Introduction**

- Welcome and remind participants that the focus groups will be recorded, their right to withdraw from the focus group, confidentiality, and data anonymisation
- Introductions (ice breaking, each one states their name and why they are interested in this research)
- Re-state the purpose of the focus groups (Researcher gives an overview of Bradford’s Living Well Faith Settings Programme, the Bradford madrasahs’ experiences of the programme and some national obesity statistics in children and in ethnic minority groups)

**Children’s health and physical activity in Bristol**

1. How **active** do you think the children in your community are? What factors do you think influence this behaviour?
2. What about their **diet**? What factors do you think influence Children’s diet?
3. What **current/previous physical activity/healthy diet programmes** delivered outside of the Madrasahs setting have benefited children from the Muslim community in Bristol?

- What are your experiences of these programmes?
- Have these programs been linked up with Madrasas before?
- What worked/didn’t work about this, or if they haven’t, are there any reasons for this? [Explore: some mosques run some physical activity programs like football competitions among mosques]

**How Madrasahs operate in Bristol**

1. How many **Madrasahs** are there in Bristol, and what are their denominations? Do they differ by ethnicity or background?

- Are they well-attended?
- How many days do they operate? Weekdays/weekend days?
- What is the typical child’s age range?
- What do you think of online lessons?
- Are there any challenges Madrasahs face in delivering their work?

1. What are your views on **mosques delivering health promotion campaigns**? How do they describe the response and the engagement of the Muslim community to health campaigns delivered via mosques?

**The Bradford programme’s translation into Bristol**

1. What are your **thoughts** on the programme?
2. How could we apply this programme in Bristol?
3. Can you identify any **potential barriers** that might need to be considered when applying this programme in Bristol?
   - - What are these barriers? (Explore inclusivity, gender differences, geographical location, and acceptability)
     - How would we potentially overcome these barriers?
4. How do you think this programme could align with the Madrasahs’ settings in Bristol?
5. What **resources** do you think we would need?
6. Who do you think could be **responsible for delivering** this programme in the madrasahs?
7. How do you feel about **contributing** to the implementation of the programme? How could you contribute to this programme?
8. What sort of **engagement** do you think the programme could reach in Bristol? How do we increase the engagement? [explore in terms of mosques' participation, community engagement, communication approach]
