# Supplementary material for: Healthy Madrasas: a qualitative study using normalisation process theory and co-production approach to explore translation of a childhood obesity prevention intervention in Islamic religious settings from one UK city to another
Source: BMJ Open. 2026 Jun 29;16(6):e115161. doi: 10.1136/bmjopen-2025-115161 (PMC13331203; doi:10.1136/bmjopen-2025-115161)
Supplement: Supplementary file 3 [file bmjopen-2025-115161.reviewer_comments.pdf]

PEER REVIEW HISTORY

BMJ Open publishes all reviews undertaken for accepted manuscripts. Reviewers are asked to complete a checklist review form and are provided with free text boxes to elaborate on their assessment. These free text comments are reproduced below.

ARTICLE DETAILS

Title (Provisional)

Healthy Madrasas: Healthy Madrasas: A qualitative study using Normalisation Process Theory and co-production approach to explore translation of a childhood obesity prevention intervention in Islamic Religious Settings from one UK city to another

Authors

Cawley, Elin; Jago, Russell; Baig, Samina; Dogra, Sufyan Abid; Haque, Zia; Hamilton-Shield, Julian; Barber, Sally Elizabeth; Al Husein, Nour

---

VERSION 1 - REVIEW

Reviewer 1

Name Lazorick, Suzanne

Affiliation Department of Public Health, Brody School of Medicine, East Carolina University, Public Health

Date 06-Mar-2026

COI None

The article 2025-115161, Healthy Madrasas: Stakeholder perceptions of the feasibility

of translating a childhood obesity prevention intervention in

Islamic Religious Settings from one UK city to another, presents findings of a qualitative study regarding adapting from one city to another an intervention for childhood obesity targeting Islamic communities. I commend the study group on the intervention design and work to disseminate to broader settings. The paper is clear and well-written, and the methods have been executed appropriately. Given the prevalence of obesity and the importance of maximizing effectiveness of interventions by tailoring to local settings and specific populations, the methods and findings are likely to be of interest more generally beyond the specific religion or cities included. Several suggestions are provided in the section for the authors to strengthen the paper for better understanding for the readers.

Specific comments/edits:

Title

Is “feasibility” the most accurate term? The methods seemed to elicit information beyond feasibility and moved to strategies for implementation/adaptation. Suggest review and revise as indicated per the study intent, methods and conclusions.

Abstract:

Edit participant section to clarify #s and % by sex in each component- e.g. workshop (n= , x % F), 7 focus groups (n= , x% F), and 1 interview (F)

Intro

Review te final statement about the purpose of the study to make sure it encompasses the full intent. From comments in other sections it seems there may have been more aims.

Methods

Make sure section headings are appropriate for the content included. Suggest moving up information about the collaboration wit the ambassadors and expand description to fully represent their role—as it seams they contributed to design, recruitment and as part of the research team (facilitating focus groups).

line 137, likely for accuracy need to add – “and interviews “ with parents; also if surveys/questionnaires were also part of data collection- include here as part of study design.

line 146-149, sentence “This guidance…” and lines 154-156 “In this study…” do not fit in this section about the framework which guided design of the workshop and study. If aim statement here was part of the overall objective then it needs to go in the final paragraph of the intro.

Provide time period- from workshop to completion of all FGs/interviews (wasit over a week or so, months?); and more basics about the workshop—delivered over xx hrs; were FGs designed to follow from (sequential) and add to the workshop? Or were FGs concurrent with the workshop? E.g line 172- The x hour workshop was split into four, consecutive x minute sections. Perhaps for each (workshop and focus groups/interview) present the info from design, to general /format, to specific content/delivery. The reader should be able to understand enough detail to replicate the process if desired.

line 186- Pls provide more info about the focus groups- design and execution. How was focus-group guide developed (by who); who led the FGs (move up info about this that is in lines 223-36), trained how? How long was each? And note (here or in participant section) that 1 planned “group” ended up being an interview due to a single participant, and 1 only had 2 participants. Were there plans to achieve saturations? How was # of groups determined?

line 196. Participant section- start with sentence line 210, “A purposive…” Then, Workshop participants included… Focus Groups/Interview participants included…

line 206-208 revise opening of the paragraph for clarity- “In order to help recruit the target participants and facilitate productive discussions, we enlisted help of representatives from the community called Health Research Ambassadors….”. Alternatively- as per general comment above—include a section that describes the contribution from and partnership with this group.

Line 219- email or phone call from who? Ambassador or study staff

lines 223-36- belong in different section (not recruitment)

line 241- accuracy checking by who?

Line 243-45, please revise for accuracy how the software was used. Typically, qualitative software does not “analyze” transcripts, rather software is used for data/code organization which supports thematic analyses that are conducted, and interpreted, by the researchers.

Section starting line 256- use of community group/ambassadors can be better described; and would be better placed earlier in methods — given the role that extended form study design, to recruitment, and then In the workshops and focus groups.

Results

Recruitment efforts- how many refused, dropped out?

Table 2 and 3- display differently to summarize characteristics and NOT list as individuals

(e.g. Overall split by sex; then subgroups for workshop; for focus groups- all F.

line 648- all were F

Table 4- This appears out of context- comparison of the 2 sites/cities; omit, or may belong earlier (in intro or methods) where describing the need for formative work to plan for the new setting.

Reviewer 2

Name Guimarães, Nathalia

Affiliation Observatório de Pesquisa em Epidemiologia, Nutrição e Saúde (OPeNS), Belo Horizonte, Brazil

Date 06-Mar-2026

COI None

The study addresses an important and underexplored topic, namely the feasibility of translating a community-based childhood obesity prevention intervention from one Islamic religious setting to another. The participatory approach and engagement with community stakeholders represent valuable strengths, particularly given the importance of culturally appropriate interventions in minority populations. However, several methodological and

analytical issues limit the robustness of the findings and should be addressed to strengthen the manuscript.

First, the methodological description raises some conceptual and procedural concerns. The authors describe the study as employing “multiple methods”, yet the data collection appears to rely exclusively on qualitative techniques (a co-production workshop and focus groups). While combining different qualitative techniques can enrich data collection, the terminology “multiple methods” may be misleading, as it is often interpreted as referring to mixed-methods approaches that integrate qualitative and quantitative components. The authors should clarify the rationale for this terminology or more precisely describe the study as a qualitative participatory design using multiple qualitative data sources.

Second, the sampling strategy and composition of participants raise concerns regarding representativeness and diversity of perspectives. The study includes 34 participants across one workshop and eight focus groups; however, the focus groups were composed exclusively of women. This gender imbalance limits the range of perspectives captured, particularly considering that parental roles, decision-making, and engagement in religious institutions may differ between mothers and fathers in many Muslim communities. The absence of male caregivers may therefore restrict the comprehensiveness of the findings and should be more critically acknowledged and discussed.

Relatedly, the size of the focus groups appears to be considerably smaller than what is typically recommended for this method. According to the manuscript, groups ranged from one to four participants. Focus groups are generally designed to stimulate interaction and collective discussion, and very small groups (especially those with one or two participants) resemble individual interviews rather than group discussions. This limitation may have reduced the richness of the interactive dynamics that focus groups are intended to capture. The authors should provide a stronger justification for maintaining these sessions as focus groups and discuss how this limitation may have influenced the data generated.

Another issue relates to the representativeness of the communities included. Although the study aims to explore the translation of an intervention to a new city with a diverse Muslim population, most participants appear to be of South Asian origin. Given that Bristol’s Muslim population includes a wider range of ethnic backgrounds (e.g., Somali and other African communities), the perspectives captured may not fully reflect the diversity of the target population. This limitation may affect the transferability of the findings and should be discussed more explicitly.

In terms of data analysis, the manuscript states that thematic analysis was conducted using NVivo and guided by the constructs of Normalisation Process Theory (NPT). While the use of a theoretical implementation framework is appropriate, the description of the analytical process lacks important methodological detail. For example, the manuscript does not clearly report how many researchers were involved in coding, whether coding was conducted independently, how disagreements were resolved, or whether any strategies were used to

enhance analytical rigor (such as reflexive discussions, audit trails, or triangulation). Providing greater transparency regarding these procedures would improve the credibility and trustworthiness of the qualitative analysis.

Regarding the results, the findings are primarily organized around three NPT constructs and largely highlight positive perceptions of the intervention and its relevance within the community. While this is useful, the analysis appears somewhat descriptive and could benefit from deeper critical engagement with the data. For instance, the manuscript would be strengthened by more detailed exploration of tensions, divergent views, or potential resistance to implementation. The predominance of supportive perspectives may partly reflect recruitment through existing community networks, which may have favored individuals already engaged with health initiatives. This possibility should be considered when interpreting the findings.

Furthermore, although the stated aim is to explore the feasibility of translating the intervention from Bradford to Bristol, the results provide relatively limited discussion of how contextual differences between the two settings might influence implementation. A more explicit comparison of contextual factors—such as community structure, institutional support, or demographic composition—would strengthen the contribution of the study to the literature on intervention transferability.

Finally, some of the recommendations emerging from the findings remain relatively general (e.g., the need for partnerships, culturally appropriate materials, and community engagement). While these points are valid, the manuscript would benefit from a more operational discussion of how such elements could be implemented in practice, particularly within madrasa settings.

VERSION 1 - AUTHOR RESPONSE

Reviewer: 1
Dr. Suzanne Lazorick, Department of Public Health, Brody School of Medicine, East Carolina University
Comments to the Author:

The article 2025-115161, Healthy Madrasas: Stakeholder perceptions of the feasibility of translating a childhood obesity prevention intervention in Islamic Religious Settings from one UK city to another, presents findings of a qualitative study regarding adapting from one city to another an intervention for childhood obesity targeting Islamic communities. I commend the study group on the intervention design and work to disseminate to broader settings. The paper is clear and well-written, and the methods have been executed appropriately. Given the prevalence of obesity and the importance of maximizing effectiveness of interventions by tailoring to local settings and specific populations, the methods and findings are likely to be of interest more generally beyond the specific religion or cities included. Several suggestions are

provided in the section for the authors to strengthen the paper for better understanding for the readers.

We thank the reviewer for their careful and generous review of our manuscript. We are grateful for the positive assessment of the intervention design, the clarity of the paper, and the methodological approach adopted. We particularly appreciate the recognition of the broader relevance of the study. We value the reviewer's constructive suggestions and have addressed each point in detail below.

Specific comments/edits:

Title

Is "feasibility" the most accurate term? The methods seemed to elicit information beyond feasibility and moved to strategies for implementation/adaptation. Suggest review and revise as indicated per the study intent, methods and conclusions.

We thank the reviewer for this helpful comment regarding the use of term "feasibility" and agree the study extends beyond an assessment of feasibility to encompass stakeholder perceptions of adaptation and contextual consideration. In response, we have revised the title to: "Healthy Madrasas: A qualitative study using Normalisation Process Theory and co-production approach to explore translation of a childhood obesity prevention intervention in Islamic Religious Settings from one UK city to another"

Abstract:

Edit participant section to clarify #s and % by sex in each component- e.g. workshop (n= , x % F), 7 focus groups (n= , x% F), and 1 interview (F)

In response to the reviewer comment, we have revised the section for clarity. The text now reads: "Data were collected through one co-production workshop (n=15, 47% Female) and eight focus groups (n=19, 100% female), two of which were conducted as individual interviews due to single attendance (n=1, 100% Female)"

Intro

Review the final statement about the purpose of the study to make sure it encompasses the full intent. From comments in other sections it seems there may have been more aims.

We have added at the end of the introduction "Specifically, we sought to identify barriers and co-produce recommendations to the integration of an intervention 'The Healthy Madrasas Programme' into madrasas settings in Bristol."

Methods

Make sure section headings are appropriate for the content included. Suggest moving up information about the collaboration with the ambassadors and expand description to fully represent their role—as it seams they contributed to design, recruitment and as part of the research team (facilitating focus groups).
We thank the reviewer for this helpful suggestion. In response, The *PPEI* section has been moved to follow the Study Design and Theoretical Framework section. The information about collaboration with Health Ambassadors has also been relocated to this section, ensuring all related content is consolidated in one place and collectively explains the role of the health ambassadors.

line 137, likely for accuracy need to add – "and interviews " with parents; also if surveys/questionnaires were also part of data collection- include here as part of study design.
In response, we have revised the text as follows: "along with focus groups and interviews involving parents of children from the Muslim community. Participants were additionally asked to complete a brief questionnaire capturing key demographic information"

line 146-149, sentence "This guidance…" and lines 154-156 "In this study…" do not fit in this section about the framework which guided design of the workshop and study. If aim statement here was part of the overall objective then it needs to go in the final paragraph of the intro.

We thank the reviewer for this helpful observation. In response, we have removed the sentence "This guidance was appropriate for this work as one of the aims of the workshop and focus group discussions were to identify and discuss potential barriers and solutions in the uptake of the Healthy Madrasas Programme in the Bristol context (Table 1)" and "In this study, we aimed to identify the barriers to the integration of an intervention prototype, 'the Bradford model', into madrasas in Bristol."

Instead we have added a clarifying sentence at the end of *Study Design and Theoretical Framework* section: "Table 1 illustrates how the study design aligns with O'Cathain's six steps of co-production and maps onto the NPT constructs"

Provide time period- from workshop to completion of all FGs/interviews (was it over a week or so, months?); and more basics about the workshop—delivered over xx hrs; were FGs designed to follow from (sequential) and add to the workshop? Or were FGs concurrent with the workshop?
We have added the following text at the beginning of the *Study Procedures* section to clarify the timing and the sequencing of data collection: "Data collection was conducted between November 2024 and June 2025. The co-production workshop took placed in November 2024 and was followed by initial analysis to inform the design and the content of the focus group discussions. Based on advice from the Health Ambassadors regarding participants' availability

during public, cultural and school holidays, the focus groups were conducted between April 2025 and June 2025."

E.g line 172- The x hour workshop was split into four, consecutive x minute sections. Perhaps for each (workshop and focus groups/interview) present the info from design, to general /format, to specific content/delivery. The reader should be able to understand enough detail to replicate the process if desired.
We thank the reviewer for this helpful suggestion. In response, A section titled "study procedure" was created to outline the design of the topic guides and to describe the structure and timing of both the workshop and the focus groups.

line 186- Pls provide more info about the focus groups- design and execution. How was focus-group guide developed (by who); who led the FGs (move up info about this that is in lines 223-36), trained how? How long was each? And note (here or in participant section) that 1 planned "group" ended up being an interview due to a single participant, and 1 only had 2 participants. Were there plans to achieve saturations? How was # of groups determined?
We thank the reviewer for this helpful comment. In response we have expanded the *Study Procedure* section to provide more details, as follows: "Focus group discussions were conducted online and facilitated by researchers (NA, EC) or Health Ambassadors (SB). Both EC and SB were trained by NA in the facilitation and delivery of the focus groups. A semi-structured topic guide was used to explore parents' perceptions of their children's health and physical activity, current physical activity and healthy diet programmes available in Bristol, the current Madrasas' context in Bristol and the transferability of Bradford's program into Bristol (Supplementary file 2). The topic guide was developed iteratively by the research team, guided by the principles of the NPT, and reviewed by our Health Ambassador for clarity and appropriateness. Additionally, discussion points arising from each focus group were incorporated into the topic guide to be explored in subsequent focus groups. At the start of each focus group, the researcher delivered a 10-minute presentation on the research topic to provide context. Participants were also shown a short video outlining the aims and the reach of the Bradford programme. The focus groups lasted an average of 64±16 minutes, excluding time spent on introductions and video viewing. Due to last-minute participant withdrawals, or insufficient enrolment for some sessions, focus groups 4 and 5 were conducted with a single participant and therefore ran as individual interviews. Focus groups 1 and 3 each included two participants"

line 196. Participant section- start with sentence line 210, "A purposive…" Then, Workshop participants included… Focus Groups/Interview participants included…
Suggestion has been incorporated.

line 206-208 revise opening of the paragraph for clarity- "In order to help recruit the target participants and facilitate productive discussions, we enlisted help of representatives from the community called Health Research Ambassadors….". Alternatively- as per general comment above—include a section that describes the contribution from and partnership with this group.
We thank the reviewer for this helpful suggestion. Information describing the role of the Health ambassadors has been moved earlier in the Methods and incorporated into the *Patient and Public Involvement and Engagement* (PPEI) section. This section has now been repositioned to follow the study design, ensuring that the partnership with the Health Ambassadors is explained early and consolidated in one place.

Line 219- email or phone call from who? Ambassador or study staff
We have revised the text to clarify that interested individuals who filled in the questionnaire were followed up by the research team. We have also reorganised this section to improve clarity, as follows: "Our health ambassadors advertised the study via email, in person, or through community digital communication networks (WhatsApp) to the target communities and distributed the study's invitation letter or poster, both of which included a link to a short demographic and contact details questionnaire hosted on Online Survey platform (33) (an online survey platform tool used for Academic Research and Public Sector organisations). The demographic questionnaire collected data on gender and ethnicity, as well as which stakeholder groups participants aligned with. For focus groups, we also asked parents whether their child/children attended a madrasa or online Islamic classes. This information was used to check their eligibility for the study. Interested individuals who filled in the questionnaire were followed up by the research team via email or phone, provided with a participant information sheet, and invited to sign a consent form. Written consent was obtained from each participant either in person or online by completing a consent form hosted on Online Surveys."

lines 223-36- belong in different section (not recruitment)
We thank the reviewer for this helpful comment. We have retained the information relating to the demographic questionnaire within the recruitment section, as it was also used to confirm participants' eligibility for the study. The remaining content has been relocated to a new section titled 'Study Procedures' to improve clarity.

line 241- accuracy checking by who?
We have revised the text as follows: "The workshop was audio-recorded, transcribed by a transcribing company approved by the University of Bristol, and anonymised by the research team (EC). The focus groups took place online using Microsoft Teams; therefore, Teams' dictation software was utilised to transcribe the focus groups. Accuracy checking of the focus groups transcripts and anonymisation were carried out by researcher EC, who listened to each recording and verified the transcripts' accuracy."

Line 243-45, please revise for accuracy how the software was used. Typically, qualitative software does not “analyze” transcripts, rather software is used for data/code organization which supports thematic analyses that are conducted, and interpreted, by the researchers.
We thank the reviewer for this helpful clarification. We have revised the wording as follows: “Transcripts were uploaded to NVivo qualitative data software to support data and codes organisation while conducting analysis. Data were analysed thematically using the guidelines described by Braun and Clarke (34)”

Section starting line 256- use of community group/ambassadors can be better described; and would be better placed earlier in methods — given the role that extended form study design, to recruitment, and then In the workshops and focus groups.
We thank the reviewer for this helpful suggestion. In response, we have moved this section earlier in the methods and was have also expanded the description to clarify their role throughout the research process: “During the study, we sought the support of a local community organisation with deep links with members of the Muslim community. This local community organisation hosts a pilot project called the Health Research Ambassadors. The Health Research Ambassadors programme is a pilot initiative that aims to strengthen connections between researchers, organisations, and underserved communities, thereby increasing the body of research addressing health inequalities (30). Health Ambassadors are members of the public undertaking scientific work in collaboration with professional scientists. Their work often involves outreach and public engagement activities (31, 32). They also act as community activists and volunteers committed to improving the well-being of the communities they belong to. Health Research Ambassadors were recruited for the project and were representatives of the study population. Throughout the project, we sought their constant input on the study design, topic guides, recruitment strategy, data collection process, and feedback on the study findings and manuscripts. Based on their valuable feedback, we adjusted the workshop activities to be culturally appropriate, improved the clarity of our topic guides, and refined our recruitment plan.”

Results
Recruitment efforts- how many refused, dropped out?
In response to the reviewer comment, we have added further details: “All organisations or Islamic settings invited to participate in the workshop (n=15) sent representatives, with the exception of two: one did not respond, while the other responded several months later, indicating that they had missed the invitation.”
We have also expanded in the description of the focus-group recruitment findings “Thirty-five individuals completed the expression of interest from, of those nineteen participants, all females, attended eight focus groups. Reasons for dropout included illness (n=2), work or family commitments (n=2), misunderstanding of the programme’s purpose (n=2) and unknown reasons (n=6).”

Table 2 and 3- display differently to summarize characteristics and NOT list as individuals (e.g. Overall split by sex; then subgroups for workshop; for focus groups- all F.

We thank the reviewer for this helpful suggestion. In response, Table 2 and 3 have been revised to present summary characteristics of participants rather than individuals.

line 648- all were F

Yes, female participants from the workshop (apologies if I misunderstood this comment)

Table 4- This appears out of context- comparison of the 2 sites/cities; omit, or may belong earlier (in intro or methods) where describing the need for formative work to plan for the new setting.

We thank the reviewer for this helpful comment. We have revised Table 4 to more clearly present the barriers identified in this study alongside co-produced recommendations (see Table 4 in the manuscript)

Reviewer: 2

Ms. Nathalia Guimarães, Observatório de Pesquisa em Epidemiologia, Nutrição e Saúde (OPeNS), Belo Horizonte, Brazil, Federal University of Minas Gerais

Comments to the Author:

The study addresses an important and underexplored topic, namely the feasibility of translating a community-based childhood obesity prevention intervention from one Islamic religious setting to another. The participatory approach and engagement with community stakeholders represent valuable strengths, particularly given the importance of culturally appropriate interventions in minority populations. However, several methodological and analytical issues limit the robustness of the findings and should be addressed to strengthen the manuscript.

We thank the reviewer for their thoughtful and constructive feedback. We appreciate the recognition of the importance of the topic and the strengths of the approach adopted in this study. In response to the points raised, we have revised the manuscript to strengthen the methodological transparency and to clarify the contextual considerations in relation to the intervention transferability. Detailed responses to each comment are provided below.

First, the methodological description raises some conceptual and procedural concerns. The authors describe the study as employing “multiple methods”, yet the data collection appears to rely exclusively on qualitative techniques (a co-production workshop and focus groups). While combining different qualitative techniques can enrich data collection, the terminology “multiple methods” may be misleading, as it is often interpreted as referring to mixed-

methods approaches that integrate qualitative and quantitative components. The authors should clarify the rationale for this terminology or more precisely describe the study as a qualitative participatory design using multiple qualitative data sources.

We thank the reviewer for this thoughtful comment and appreciate the opportunity to clarify our use of the terminology. We have used the term "multiple methods" to describe the different approaches that we used to collect data. We believe that this is consistent with widely used terms and approaches in the field. For example:

- Morse, J. M. (2003). Principles of mixed methods and multi-method research design. In Tashakkori & Teddlie (Eds.), Handbook of Mixed Methods in Social and Behavioral Research (pp. 189–208). SAGE.
-Cook, N., McConnell, D., Teeling, S.P. (2021). Multiple and Mixed Methods Research. In: Dewing, J., McCormack, B., McCance, T. (eds) Person-centred Nursing Research: Methodology, Methods and Outcomes. Springer, Cham. https://doi.org/10.1007/978-3-030-27868-7_11
-Anguera, M.T., Blanco-Villaseñor, A., Losada, J.L. et al. Revisiting the difference between mixed methods and multimethods: Is it all in the name?. Qual Quant 52, 2757–2770 (2018). https://doi.org/10.1007/s11135-018-0700-2

With that in mind, we respectfully suggest that the term "multiple methods" is not inherently misleading. In our opinion, neither these references provided, nor the wider methodological literature, indicate that the use of the use of this term is contingent on the inclusion of a quantitative component. Rather, multiple-method research is commonly understood to refer to the use of more than one method, qualitative or quantitative, within a single study. As articulated in these resources we cited above, multiple-method designs involve the use of "two complete methods" each of which is "complete in itself" (regardless whether they are qualitative or quantitative), with findings subsequently brought together through synthesis and interpretation. By contrast, mixed methods is a specific subset uses qualitative and quantitative methods but defined by integration of data sets leading to generate Meta-themes.

That said, we appreciate the reviewer's suggestion and agree that greater specificity may ensure clarity for readers from diverse methodological background. We are therefore happy to revise the wording to describe the study as "qualitative participatory design using multiple methods".

Second, the sampling strategy and composition of participants raise concerns regarding representativeness and diversity of perspectives. The study includes 34 participants across one workshop and eight focus groups; however, the focus groups were composed exclusively of women. This gender imbalance limits the range of perspectives captured, particularly considering that parental roles, decision-making, and engagement in religious institutions may differ between mothers and fathers in many Muslim communities. The absence of male caregivers may therefore restrict the comprehensiveness of the findings and should be more critically acknowledged and discussed.

We thank the reviewer for raising this important point. We agree that the absence of male caregivers in the focus groups limits the range of the parental perspectives captured. This issue is explicitly acknowledged and critically discussed in the limitation section of the manuscript. As noted, while the workshop was well attended by both men and women, including representatives of Islamic organisations, roles more commonly held by men (some of them are fathers themselves), no male caregivers ultimately participated in the parent focus groups, despite offering multiple sessions at different times to accommodate varying working patterns. We reflect on this outcome in relations to existing gendered divisions of caregiving labour and cultural norms within many Muslim communities, where parental day-to-day responsibilities more frequently fall to women. In this sense, the composition of the focus groups is not only a methodological limitation but also reflects the social realities shaping parental engagement.
In light of the reviewer's comment, we edited the limitations section to "although the workshop was well attended by both men and women, no male participants took part in the focus groups, despite offering various time slots to accommodate different working patterns. The absence of male caregivers in the focus groups limits the range of the parental perspectives captured and may restrict the comprehensiveness of the findings. However, this outcome may be due to existing traditional gender roles and cultural norms. While the workshop was aimed at representatives of Islamic organisations, roles often held by men such as Imams, the focus groups were aimed at parents. Given that the caring responsibilities traditionally fall to women, it is unsurprising that all our parent participants were female. In this sense, the composition of the focus groups is not only a methodological limitation but also reflects the social realities shaping parental engagement. Future research should therefore employ targeted and gender-sensitive engagement approaches to support the inclusion of male caregivers. For example, the Bradford study successfully recruited parents of both genders, likely due to the presence of a male researcher."

Relatedly, the size of the focus groups appears to be considerably smaller than what is typically recommended for this method. According to the manuscript, groups ranged from one to four participants. Focus groups are generally designed to stimulate interaction and collective discussion, and very small groups (especially those with one or two participants) resemble individual interviews rather than group discussions. This limitation may have reduced the richness of the interactive dynamics that focus groups are intended to capture. The authors should provide a stronger justification for maintaining these sessions as focus groups and discuss how this limitation may have influenced the data generated.

We thank the reviewer for this thoughtful comment and agree that small group sizes may limit the interactive dynamics typically associated with focus groups. We acknowledge that some sessions included fewer participants than conventionally recommended due to last minute dropout or insufficient enrolment. This deviation from ideal focus group size arose from the

practical and ethical realities of conducting research with communities that are often described as underserved, hard to reach and for whom participation in research activities maybe unfamiliar or approached with caution. In this context, cancelling under-subscribed sessions risked excluding already recruited participants and would further exacerbate barriers to engagement. We therefore prioritised inclusivity and accessibility by proceeding with the scheduled sessions.
In light of the reviewer's comment, we have clarified in the manuscript that sessions with one participant were treated as individual interviews:
In the abstract "Data were collected from one workshop (n=15, 47% F) and eight focus groups (n=19, 100%), of which two were conducted as interviews (n=1, 100% F)."
In the study design "…along with focus groups and interviews involving parents of children from the Muslim community"
In the methods section "Due to last-minute participant withdrawals, or insufficient enrolment for some sessions, focus groups 4 and 5 were conducted with a single participant and therefore ran as individual interviews. Focus groups 1 and 3 each included two participants."

We also added to the limitations section "Additionally, due to recruitment challenges within hard-to-reach communities, several focus groups were under-subscribed and ran with one or two participants (with single-participant sessions conducted as individual interviews). In these occasions, cancelling these sessions may risk excluding already recruited participants and would further exacerbate barriers to engagement. We therefore prioritised inclusivity and accessibility by proceeding with the scheduled sessions. Nevertheless, these sessions generated rich, in-depth accounts aligned with the study aims. We recommend that future studies in similar contexts to employ hybrid formats to balance methodological ideals with feasibility."

Another issue relates to the representativeness of the communities included. Although the study aims to explore the translation of an intervention to a new city with a diverse Muslim population, most participants appear to be of South Asian origin. Given that Bristol's Muslim population includes a wider range of ethnic backgrounds (e.g., Somali and other African communities), the perspectives captured may not fully reflect the diversity of the target population. This limitation may affect the transferability of the findings and should be discussed more explicitly.
We thank the reviewer for raising this important point. This issue is explicitly acknowledged in the limitations section where we noted the lower Somali participation which may reflect less established community connections. We recognise that this issue may limit the transferability of findings to the wider Muslim communities in Bristol. That said, participation from the Somali community did take part in both the workshop and the focus groups (5 participants in total). In light of the reviewer's comment, we have revised Table 2 to more clearly present the ethnic diversity of participants, including Pakistani, Indian, Bangladeshi, Somali, Arab and other Asian backgrounds which reflects the range of the Muslim communities in Bristol.

In terms of data analysis, the manuscript states that thematic analysis was conducted using NVivo and guided by the constructs of Normalisation Process Theory (NPT). While the use of a theoretical implementation framework is appropriate, the description of the analytical process lacks important methodological detail. For example, the manuscript does not clearly report how many researchers were involved in coding, whether coding was conducted independently, how disagreements were resolved, or whether any strategies were used to enhance analytical rigor (such as reflexive discussions, audit trails, or triangulation). Providing greater transparency regarding these procedures would improve the credibility and trustworthiness of the qualitative analysis.

We thank the reviewer for this helpful comment. In light of the reviewer's comment, we have also expanded in the analysis section to improve clarity and methodological details: "As the study adopted a multiple methods qualitative design, we (NA, EC) were able to apply methodological triangulation within methods (the combination of two or more data collections, in this case qualitative methods, to examine the same phenomena) (38). Following the co-production workshop, which brought together multiple stakeholders involved in madrasa delivery and health promotion for the target populations, we conducted (as described above) thematic analysis to identify codes and themes. This analysis informed the discussions of the subsequent focus groups, allowing for a deeper understanding for the identified codes and themes. After completion of the focus groups, a further thematic analysis was undertaken on the focus groups data which generated additional codes and themes. The analyses from both data sets were then brought together, enabling the development of overarching themes (39). In this case, the two qualitative methods generated complementary data and strengthen the credibility of the findings though within-method triangulation"

Regarding the results, the findings are primarily organized around three NPT constructs and largely highlight positive perceptions of the intervention and its relevance within the community. While this is useful, the analysis appears somewhat descriptive and could benefit from deeper critical engagement with the data. For instance, the manuscript would be strengthened by more detailed exploration of tensions, divergent views, or potential resistance to implementation. The predominance of supportive perspectives may partly reflect recruitment through existing community networks, which may have favored individuals already engaged with health initiatives. This possibility should be considered when interpreting the findings. Furthermore, although the stated aim is to explore the feasibility of translating the intervention from Bradford to Bristol, the results provide relatively limited discussion of how contextual differences between the two settings might influence implementation. A more explicit comparison of contextual factors—such as community structure, institutional support, or demographic composition—would strengthen the contribution of the study to the

literature on intervention transferability.

We thank the reviewer for this comment and respectfully disagree that the analysis predominantly presents supportive or purely descriptive findings, or it lacks engagement with tensions, divergent views, or potential barriers to implementation. In fact, a central contribution of this study lies in its identification of multiple barriers, and contextual challenges that need to be addressed for a successful transfer and implementation of the intervention in Bristol.

Across the Normalisation Process Theory (NPT) constructs, our findings explicitly document areas of limited coherence and potential barriers, particularly in relation to cultural and religious conservatism affecting female participation, perception of appropriate activities for girls (see theme 3, overcoming old traditions) and concerns about the use of mosques and madrasas as spaces for physical activity. Parents described some madrasas environment as intimidating or overly strict, with teaching styles and culturally specific norms that maybe exclusionary of off-putting for some children and families (theme 2, Current Madrasas' structure). Female participants further articulated tensions around gender power dynamics, reporting that women's voices and initiatives were sometimes marginalised, that communication pathways were ineffective (theme 3, overcoming old traditions, communication). In relation to collective action, the findings highlight significant practical and structural barriers, including uncertainty around leadership, the need for a neutral and inclusive steering committee (theme 3, Leadership and collective action), limited organisational capacity within Bristol Islamic institutions and lack of trust in some funding sources perceived to conflict with Islamic values. These concerns were compounded by anxieties around sustainability, with several participants referencing previous initiatives that failed due to insufficient resources and volunteer fatigue (theme 3, Structural barriers, resources and sustainability). We also explicitly engage with contextual differences between Bradford and Bristol, which is a core focus of the results (theme 3, The differences between Bradford and Bristol). Participants contrasted Bradford's larger, more established Muslim population, with greater access to volunteers, infrastructure and services, with Bristol's smaller, more ethnically diverse and geographically dispersed Muslim communities. These contextual distinctions were identified as critical for feasibility, particularly in relation to community buy-in, leadership formation, communication strategies and equitable reach across diverse groups.

Regarding the recruitment through community networks, we acknowledge that this might have facilitated engagement from individuals who are already engaged with health initiatives. However, the diversity of the perspectives presented including the barriers that both parents and religious leaders expressed, suggests that the data captured a board and reflexive range of views. Moreover, the co-production approach was intentionally chosen to surface both barriers and facilitators at an early stage of implementation planning, which is successfully

achieved.

We would also like to underline the importance of remaining faithful to the data and participants' voices. While participants were generally positive about the programme, they clearly identified a range of practical, cultural and structural barriers to successful implementation, which they framed as issues requiring careful consideration rather than opposition to the programme itself. Had participants expressed these barriers as insurmountable, a more critical presentation would have been appropriate. We were therefore cautious not to impose academic labels such as "resistance" or "divergence" where these did not reflect participant's intent, as doing so risks reframing their accounts to satisfy academic convention rather than accurately representing their perspectives.
That said, in response to the reviewer's suggestion, we have revised Table 4 to more explicitly signpost to identified barriers, making them clearer and more concrete (see table 4 in the manuscript)

Finally, some of the recommendations emerging from the findings remain relatively general (e.g., the need for partnerships, culturally appropriate materials, and community engagement). While these points are valid, the manuscript would benefit from a more operational discussion of how such elements could be implemented in practice, particularly within madrasa settings.

We thank the reviewer for this thoughtful critique. The study was explicitly designed as early-phase, pre-implementation exploration of feasibility and transferability. At this stage, implementation science emphasises identifying contextual barriers and facilitators for adoption rather than prescribing fixed delivery models. The co-production approach adopted here was therefore intentionally used to surface stakeholders priorities and implementation requirements, which presented in the manuscript. These findings provide a clear foundation for subsequent phases of planning and pilot testing. That said, in response to the reviewer's suggestion, we have revised Table 4 to more clearly present the co-produced recommendations (see Table 4 in the manuscript)

VERSION 2 - REVIEW

Reviewer 1

Name Lazorick, Suzanne

Affiliation Department of Public Health, Brody School of Medicine, East Carolina University, Public Health

Date 31-May-2026

COI

In this revision, R1, the authors have sufficiently addressed my suggestions and those of the editor. The paper is stronger and the methods are more thoroughly described. I defer to the editor and other reviewers regarding whether additional suggestions have been sufficiently addressed.

Reviewer **2**

Name **Guimarães, Nathalia**

Affiliation **Observatório de Pesquisa em Epidemiologia, Nutrição e Saúde (OPeNS), Belo Horizonte, Brazil**

Date **17-May-2026**

COI

The authors have carefully addressed the comments raised by both reviewers and the editorial team. The revisions substantially improved the clarity, methodological transparency, and overall quality of the manuscript. In particular, the authors strengthened the description of the study design, participant recruitment, analytical procedures, and the role of Health Research Ambassadors throughout the research process. The manuscript now provides a clearer and more robust presentation of the qualitative methods employed, including important contextual details regarding focus groups, interviews, co-production activities, and thematic analysis. I recommend acceptance of the manuscript for publication.
